# Effectiveness of Canakinumab Treatment in Colchicine Resistant Familial Mediterranean Fever Cases

**DOI:** 10.3389/fped.2021.710501

**Published:** 2021-09-10

**Authors:** Burcu Bozkaya Yücel, Ozlem Aydog, Hulya Nalcacioglu, Ayşegül Yılmaz

**Affiliations:** ^1^Department of Pediatric Rheumatology, Ondokuz Mayis University Faculty of Medicine, Samsun, Turkey; ^2^Department of Pediatric Nephrology, Ondokuz Mayis University Faculty of Medicine, Samsun, Turkey; ^3^Department of Pediatric Genetics, Ondokuz Mayis University Faculty of Medicine, Samsun, Turkey

**Keywords:** canakinumab, colchicine resistant, familial mediterranean fever, treatment, amyloidosis, proteinuria

## Abstract

Anti-interleukin 1 agents are used successfully in colchicine-resistant or intolerant Familial Mediterranean Fever (FMF) patients. Sixty-five patients with FMF who received canakinumab treatment for at least 6 months due to colchicine resistance or intolerance between 2016 and 2020 in our department were retrospectively analyzed. Canakinumab treatment was given subcutaneously every 4 weeks. After completing monthly canakinumab therapy over 12 months, in patients with complete remission, the dosing interval was extended to every 1.5 months for 6 months, then every 2 months for 6 months, and finally every 3 months for a year. In patients without disease activation, canakinumab treatment was discontinued at the end of 3 years and followed up with colchicine treatment. Patients who had a flare switched to the previous dosing interval. In patients with renal amyloidosis, monthly canakinumab treatment was continued without extending the dose intervals. The mean duration of canakinumab use in our patients was 31.4 ± 10.57 months (6–52 months). The mean age at onset of symptoms was 4.65 ± 3.84 (range, 1–18) years, and the mean age at diagnosis was 5.59 ± 3.9 (range, 4–19) years. Complete remission was achieved in 57 (87.6%) and partial remission in seven (10.7%) patients. One patient was unresponsive to treatment. Canakinumab treatment was discontinued in three patients with complete remission and one patient with drug resistance. Erythrocyte sedimentation rate (ESR) (51.85 ± 15.7 vs. 27.80 ± 13.73 mm/h) and C-reactive protein (CRP) [26 (3-73) vs. 5 (1–48) mg/L] values were compared before and after canakinumab treatment in attack-free periods, a significant decrease was found after canakinumab treatment (*p* < 0.001, *p* < 0.001, respectively). Bodyweight *Z*-scores (respectively −0.80 ± 0.86 vs. −0.49 ± 0.92) were compared, similarly, a statistically significant increase after canakinumab treatment (*p* < 0.001), but no significant increase in height *Z* scores (−1.00 ± 0.88 vs. −0.96 ± 0.94) (*p* = 0.445) was detected. Four patients had FMF-related renal amyloidosis. The decrease in proteinuria with canakinumab treatment was not statistically significant (*p* = 0.068). Cervical lymphadenitis developed in one and local reactions in two patients. No severe adverse effects requiring discontinuation of canakinumab treatment were observed. Our study showed that canakinumab treatment was highly effective, well-tolerated in pediatric FMF patients, and controlled extension of the canakinumab dose interval was safe.

## Introduction

Familial Mediterranean fever (FMF) is the most common autoinflammatory disease with autosomal recessive inheritance, characterized by recurrent attacks of fever and serositis. The disease often affects ethnic groups of the Eastern Mediterranean region, and the frequency of FMF carriers among Sephardic and Ashkenazi Jews, Arabs, Italians, Armenians, and Turks is around 1/3–1/5 (1, 2).

FMF occurs due to mutations of the Mediterranean fever (*MEFV*) gene located on the short arm of chromosome 16. This gene encodes a protein made up of 781 amino acids called pyrin (also known as marenostrin). Pyrin activates caspase-1 *via* apoptosis-associated speck-like protein with a caspase recruitment domain (ASC), interacting with *NLRP3-*inflammasome, and converts pro-IL-1 into active IL-1β, the main cytokine of fever and inflammation. Gain of function mutations in the *MEFV* gene cause increased activation of the pyrin/inflammasome complex (3).

In FMF attacks, fever and serosal inflammation findings can be seen and usually last 12–72 h (4). Patients are often asymptomatic between attacks. Sometimes, subclinical inflammation continues between attacks, and amyloidosis may develop in these patients in the long term. Amyloidosis development is affected by many conditions such as ethnicity, genetic and environmental factors, and it is particularly associated with the *M694V* homozygous mutation (5).

FMF treatment aims to prevent attacks and the development of amyloidosis, and colchicine is the mainstay of the treatment. Complete remission is provided in ~75% of patients with colchicine treatment. Partial remission occurs in 15–20% of patients. Unresponsiveness or resistance to colchicine occurs in 5–10% of patients (6). In 2–10% of patients, colchicine intolerance may be observed with adverse or toxic effects. Although diarrhea and elevation of hepatic enzymes are common, leukopenia can rarely be seen. Genetic, psychogenic, hormonal, and environmental factors such as infection, drug bioavailability, gene polymorphisms in colchicine transport, and the use of drugs interacting with ABCB1 and CYP3A4 may cause colchicine resistance by altering the serum colchicine level (7).

The therapeutic blockade of IL-1β is highly effective in treating patients with FMF with colchicine resistance or intolerance. Anakinra (recombinant antagonist of the IL-1 receptor), rilonacept (human dimeric fusion protein), and canakinumab (human monoclonal antibody against IL-1β) are increasingly used in therapy (7). In a systemic literature review of 67 publications, anakinra, canakinumab, and etanercept were found to be the most frequently used biologics and anti-IL-1 therapies appear to be effective and safe in the treatment of FMF, including colchicine resistance FMF and FMF-related amyloidosis (8).

In this study, the treatment responses of patients with FMF treated with IL-1β blockade due to colchicine resistance or intolerance in Ondokuz Mayis University Medical Faculty Pediatric Rheumatology Clinic were evaluated.

## Materials and Methods

This study began after approval was obtained from the ethics committee of Ondokuz Mayis University (No: OMUKAEK 2021/1). Sixty-five patients with FMF who received canakinumab treatment for at least 6 months due to colchicine resistance or intolerance between August 2016 and August 2020 in Ondokuz Mayis University Faculty of Medicine, Department of Pediatric Rheumatology were retrospectively analyzed. Demographic and clinical characteristics, genetic mutations, comorbid conditions, and canakinumab treatment responses were evaluated in 60 (92.3%) patients with colchicine resistance and five (7.7%) patients with colchicine intolerance. A renal biopsy was performed to detect the development of amyloidosis in patients with persistent proteinuria or nephrotic syndrome.

For mediterranean fever (*MEFV*) gene analysis, DNA was isolated from 200 μL of peripheral blood samples, using QIAamp DNA Blood Mini Kit (Qiagen Inc., Hilden Germany) and stored at −20°C until the polymerase chain reaction step. Multiplex polymerase chain reaction and probe ligation reaction were performed using GML SNP detective *MEFV* kit v.1.2 (Genomed, Altendorf Switzerland) according to the manufacturer's instructions. DNA fragments were separated using capillary electrophoresis with ABI 3500XL Genetic Analyzer (Applied Biosystems Co. Ltd. California USA). Results were analyzed using the GeneMapper 5.0 software (Applied Biosystems Co. Ltd. California USA). The system allows the detection and genotyping (heterozygous or homozygous) of 18 point mutation (*E148Q, R202Q, S179I, E167D, P369S, P350R, Y471X, F479L, M694V, A744S, M680I, G632A, V727A, K695R, K695N, M694I, R761H, I692del*) of the human *MEFV* gene.

Colchicine resistance is defined as the presence of recurrent clinical episodes (≥1 attack per month over 3 months) or high acute-phase reactants (APR) unexplained by any other reasons between attacks in a patient receiving the maximum tolerable colchicine dose. Complete remission is defined as an absence of attacks and normal levels of APR after canakinumab use. Partial remission is defined as a decrease in the frequency and severity of attacks and/or partial improvement in APR after canakinumab use.

All patients used colchicine regularly for at least 3 months at the maximum tolerable dose (0.06 mg/kg/day, max 2 mg/day). Colchicine resistance or intolerance was re-evaluated using a different colchicine preparation before adding IL-1 blocker to the treatment, and if colchicine resistance or intolerance persisted, anti-IL-1 therapy was initiated. At first, seven patients started anakinra treatment, but they did not want to have daily injections, so they were switched to canakinumab treatment. Colchicine treatment was continued at tolerable doses in all patients with canakinumab treatment. Canakinumab treatment was given subcutaneously every 4 weeks at a dose of 2–4 mg/kg for patients weighing <40 kg and 150 mg for patients weighing 40 kg and over. The dose was increased in patients who developed partial remission or remained unresponsive. After completing monthly canakinumab therapy over 12 months, in patients with complete remission, the dosing interval was extended to every 1.5 months for 6 months, then every 2 months for 6 months, and finally every 3 months for a year. In patients without disease activation, canakinumab treatment was discontinued at the end of 3 years and followed up with colchicine treatment. Patients who had a flare switched to the previous dosing interval. In patients with renal amyloidosis, monthly canakinumab treatment was continued without extending the dose intervals.

Erythrocyte sedimentation rate (ESR) and C-reactive protein (CRP) values were compared before and after canakinumab treatment in attack-free periods. The normal range for ESR is 0–20 mm/h, and 0–5 mg/L for CRP.

Statistical Packages for the Social Sciences (SPSS) version 24 was used for statistical analysis. The normality in distribution of numeric variables were investigated using visual (histogram and probability diagrams) and analytic (Shapiro-Wilk) tests. The characteristics of patients' were determined using descriptive statistics. Categorical variables are represented as numbers and percentages. Parameters compatible with normal distribution were defined as mean ± standard deviations (SD), and parameters that did not fit normal distribution were defined as medium and distribution (lower-upper limit). For the comparisons between the pre- and post canakinumab treatment data of the patients', the paired samples *t*-test was used for parameters with normal distribution, and the Wilcoxon rank-sum test was used for parameters with non-normal distribution. Chi-square test was used in the analysis of qualitative data, and Fischer test in unmet test conditions. *P* < 0.05 was considered statistically significant.

## Results

Of the 65 patients included in the study, 36 (55.4%) were female, and 29 (44.6%) were male. The mean age at onset of symptoms was 4.65 ± 3.84 (range, 1–18) years, and the mean age at diagnosis was 5.59 ± 3.9 (range, 4–19) years. Forty-eight patients (73.8%) had a history of FMF in their first or second-degree relatives.

In addition to frequent clinical findings such as fever, gastrointestinal and joint involvement, 11 (16.9%) patients had hepatomegaly and/or splenomegaly, eight (12.3%) had pleuritis, three (4.6%) had erysipelas-like erythema, three (4.6%) had epididymo-orchitis, and four (6.2%) patients had renal amyloidosis.

Thirteen (20%) patients had comorbid disease: *HLA B27* negative sacroiliitis (*n* = 3), oligoarticular juvenile idiopathic arthritis (JIA) (*n* = 1), inflammatory bowel disease (IBD) (*n* = 3), IgA vasculitis (*n* = 3), systemic lupus erythematosus (SLE) (*n* = 1), and periodic fever aphthous stomatitis, pharyngitis, adenitis (PFAPA) (*n* = 2). The demographic and clinical features of the patients are given in Table 1.

**Table 1:** Demographic and clinical features of the patients.

**General features**		**Value**
No. of patients (boys/girls)		65 (29/36)
Age at onset of symptoms, mean (range) years		4.65 ± 3.84 (1–18)
Age at diagnosis, mean (range) years		5.59 ± 3.9 (4–19)
Family history, *n* (%)		48 (73.8)
Duration of canakinumab treatment, mean (range) months		31.4 ± 10.57 (6–52)
Symptoms/Signs	Abdominal pain, *n* (%)	53 (81.5%)
	Fever, *n* (%)	52 (80%)
	Arthralgia, *n* (%)	35 (53.8)
	Chest pain, *n* (%)	8 (12.3%)
	Hepatomegaly/splenomegaly, *n* (%)	11 (16.9%)
	Erysipelas-like erythema, *n* (%)	3 (4.6%)
	Epididymo-orchitis, *n* (%)	3 (4.6)
Comorbidities	Sacroiliitis	3 (4.6%)
	Oligoarticular juvenile idiopathic arthritis	1 (3.1%)
	Inflammatory bowel disease	3 (4.6%)
	IgA vasculitis	3 (4.6%)
	Systemic lupus erythematosus	1 (1.5%)
	Periodic fever, aphthous stomatitis, pharyngitis, and adenitis (PFAPA)	2 (3.1%)

*MEFV* gene mutation analysis was performed in all patients. Fifty-one (78.4%) patients were detected as *M694V* homozygous; five (7.7%) as *M694V/M680I* compound-heterozygous, four (6.1%) as *M694V* heterozygous, one (1.5%) as *M694V/V726A* compound-heterozygous, three (4.6%) as *V726A* heterozygous, and one (1.5%) patients as *M680I* heterozygous mutations. *MEFV* gene analysis of the patients is given in Table 2.

**Table 2:** MEFV- gene analysis in patients receiving canakinumab.

**MEFV- gene analysis**	***N***	**%**
M694V/M694V	51	78.4
M694V/M680I	5	7.7
M694V heterozygous	4	6.1
M694V/V726A	1	1.5
V726A heterozygous	3	4.6
M680I heterozygous	1	1.5

In a colchicine-resistant patient with *V726A* heterozygous mutation, a heterozygous *V377I* mutation in the *MVK* gene was also found. One patient with a *M694V* homozygous mutation developed hypoparathyroidism while receiving colchicine and canakinumab treatment, but no autoimmunity was detected in the etiology.

Anakinra was used initially in seven of 65 patients for IL-1 blockade. Although the patients were in complete remission with anakinra, they did not want to continue their daily injections, so they switched to canakinumab treatment 3 months after anakinra treatment.

The mean duration of canakinumab use in our patients was 31.4 ± 10.57 (range, 6–52) months. During the study, canakinumab treatment could be discontinued in nine patients due to remission, but reactivation was observed in six patients, and canakinumab treatment was restarted.

When ESR (51.85 ± 15.7 mm/h vs. 27.80 ± 13.73 mm/h) and CRP [26 (3–73) vs. 5(1–48) mg/L)] values were compared before and after canakinumab treatment in attack-free periods, a significant decrease was found after canakinumab treatment (*p* < 0.001, *p* < 0.001, respectively).

When the bodyweight *Z* scores (respectively −0.80 ± 0.86 vs. −0.49 ± 0.92) were compared, similarly, a statistically significant increase was detected after canakinumab treatment (*p* < 0.001), but there was no significant increase in height *Z* scores (−1.00 ± 0.88 vs. −0.96 ± 0.94) (*p* = 0.445).

Renal biopsy was performed in four patients with persistent proteinuria or nephrotic syndrome, and they were diagnosed as having renal amyloidosis. *M694V* homozygous mutation was detected in all patients with renal amyloidosis. In all four patients, canakinumab treatment was initiated concurrently with the diagnosis of amyloidosis. The first patient was diagnosed as having renal amyloidosis secondary to FMF when he presented with recurrent abdominal pain and nephrotic syndrome at the age of 16 years. Although there was a significant decrease in proteinuria with colchicine and canakinumab treatment for 3 years, proteinuria persisted at the nephrotic range. The second patient was diagnosed as having FMF at the age of 5 years, and colchicine treatment was initiated. This patient did not come to hospital visits and was non-compliant with treatment. He was readmitted at the age of 12 years with proteinuria and diagnosed as having renal amyloidosis. With canakinumab treatments for 3 years, although there was a significant decrease, proteinuria persisted at the nephrotic range. The third patient presented with non-nephrotic range proteinuria at the age of 9 years and was diagnosed as having renal amyloidosis secondary to FMF. Proteinuria resolved at the end of the 1st year; he is still being followed and remains in complete remission with colchicine and canakinumab treatment after 3 years. The fourth patient was admitted with recurrent fever, abdominal pain, and proteinuria at the age of 4 years. He was diagnosed as having renal amyloidosis secondary to FMF. With colchicine and canakinumab treatments, he has been in complete remission for 2 years and is still being followed. Although there was a decrease in proteinuria with canakinumab treatment, no statistically significant difference was found (*p* = 0.068). The laboratory values of four patients with renal amyloidosis before and after canakinumab treatment are shown in Table 3.

**Table 3:** Renal parameters in FMF-related amyloidosis patients.

	**Albumin** **(g/dl)**		**Creatinine** **(mg/dl)**		**Spot urine** **protein/creatinine**		**24 h urine protein** **excretion (gr/day)**		**Duration of disease/amyloidosis**	**Duration of treatments**
	**BT**	**AT**	**BT**	**AT**	**BT**	**AT**	BT^*^	AT^*^	**Time since FMF diagnosis/Time since amyloidosis diagnosis, years**	
1	2.8	3.5	0.54	0.43	6	1,4	4	2.2	3/3	Colchicine and Canakinumab for 3 years
2	1.6	4.3	0.7	0.91	3	1.5	5	3.5	10/3	Colchicine for 10 years, Canakinumab for 3 years
3	4.4	5.1	0.57	0.72	3	0.1	1	0.1	3/3	Colchicine and Canakinumab for 3 years
4	3.8	3.8	0.2	0.3	2.2	0.5	0.4	0.2	2/2	Colchicine and Canakinumab for 2 years

*BT, Before Treatment; AT, After Treatment*.

* *p = 0.068*.

Of the 65 patients, complete remission was achieved in 57 (87.6%) and partial remission in seven (10.7%); the desired response was not achieved in only one patient. Canakinumab treatment could be discontinued in three patients who were in complete remission. Canakinumab treatment continues at varying doses and intervals in 61 (93.8%) patients.

In only one patient, canakinumab treatment was discontinued due to drug resistance, and this patient is under follow up in remission with anti-tumor necrosis factor (TNF) treatment. The only patient with canakinumab resistance was *M694V* homozygous and had comorbidity of *HLA B27* negative sacroiliitis and inflammatory bowel disease (IBD). Despite improvement in FMF attacks with monthly canakinumab treatment, his axial/peripheral joint involvement and increased APRs continued after 1 year. Switching canakinumab to anti-TNF therapy in the patient provided complete improvement in joint findings and APR values.

One of our patients, who was aged 8 years, was admitted to the emergency department with fever, joint pain, and abdominal pain lasting for 15 days. No signs of leukemic involvement or hemophagocytosis were found in bone marrow aspiration. His ferritin level was 10,929 ng/mL, CRP level was 78 mg/L, aspartate aminotransferase (AST) level was 439 U/L, and platelet count was 84,000/μL. No infectious, malignant or immunologic etiology was found and he was evaluated as having macrophage activating syndrome (MAS). His symptoms and laboratory parameters improved with pulse steroid and cyclosporine treatment. He had a second MAS attack when he was tapering steroids and stopped cyclosporine treatment 3 months after the first attack. His *MEFV* genetic analysis resulted in a *V726A* heterozygous mutation. The patient was started on colchicine, cyclosporine, and canakinumab treatments, and he recovered after treatment. Cyclosporine was stopped 6 months after initiation. He has been in complete remission for 3 years with canakinumab treatment (3 monthly) in addition to colchicine treatment.

Canakinumab treatment was generally well-tolerated in our cases, and no adverse effects were observed in 62 (95.4%) of them. In the first injections of two patients, a local reaction developed at the injection site, resolving topical antihistamine and did not reoccur in subsequent injections. One of our patients developed an infection (cervical lymphadenitis) requiring antibiotic treatment. However, no severe adverse effects requiring discontinuation of canakinumab treatment were observed in any patient.

## Discussion

In this study, the treatment responses of 65 pediatric patients with FMF who received canakinumab treatment due to colchicine resistance or intolerance were evaluated. Complete remission was achieved in 57 patients, and partial remission was achieved in seven patients; the desired response was not achieved in only one patient. Although the FMF attacks of the patient who was colchicine-resistant were controlled with canakinumab treatment, the findings of peripheral/axial arthritis, enthesitis, and increased APRs continued while the patient was also receiving methotrexate treatment. In this patient, clinical and laboratory complete remission was achieved with an anti-TNF agent. In the presence of spondyloarthropathy (SpA) or IBD accompanying FMF, as in our patient, anti-TNF agents are recommended as a treatment option (9, 10). Cherqaoui et al. reported that in patients with FMF-associated juvenile SpA, colchicine response is weaker, and these patients should be treated like with juvenile SpA (11). Babaoglu et al. stated that axial SpA and IBD should be investigated in patients with FMF who receive canakinumab treatment and do not show improvement in laboratory parameters (12). Anti-IL-6 and anti-TNF agents were also effective in colchicine-resistant or intolerant patients with FMF (13, 14).

Fifty-seven (87.6%) patients developed complete remission, and seven (10.7%) were in partial remission, two of whom had nephrotic-range proteinuria, and five had rare and mild attacks; thus they were considered as being partial remission. An *M694V* homozygous mutation was detected in six of the seven patients with partial remission. In one study, exon 10 mutations were found in all colchicine-resistant patients, and these mutations were associated with disease severity and treatment resistance (15).

In a phase 3 cluster study that examined the disease activity of 60 patients who received canakinumab treatment for 72 weeks, Özen et al. reported that the incidence of attacks was low, median CRP levels were normal, and despite serum amyloid A (SAA) levels being above average (10 mg/L), they were below the threshold value (30 mg/L). These results emphasize the potential of canakinumab treatment as a long-term treatment choice in colchicine-resistant patients with FMF (16).

A meta-analysis of 27 publications involving more than 100 patients with FMF with colchicine resistance or intolerance reported that a dramatic clinical response was obtained with a very good safety profile with anti-IL1 treatment (17). In our study, canakinumab treatment was well-tolerated, and no severe adverse effects requiring treatment discontinuation were observed.

Growth parameters may be affected by systemic and local adverse effects of proinflammatory cytokines on the growth hormone (GH)/insulin-like growth factor (IGF)-1 axis in children with chronic inflammatory disease (18). The catabolic process caused by chronic inflammation can also negatively affect growth parameters. In the study of Balci et al., no significant change was found in the growth parameters of patients who received canakinumab treatment for autoinflammatory disease (AID) (19). In our study, a statistically significant increase in body weight *Z* scores was observed with canakinumab treatment. However, the lack of a significant increase in height might be due to the late initiation of canakinumab treatment or the relatively short follow-up period.

When the 24-h urine protein values of four patients with renal amyloidosis were compared before and after canakinumab treatment in attack-free periods, there was no statistically significant difference (*p* = 0.068). Proteinuria was completely resolved in two patients, and there was a significant decrease in proteinuria in the other two patients. In all four patients, serum albumin levels returned to normal and renal functions were preserved during the study. Ugurlu et al. followed 40 adult patients with FMF-related amyloidosis receiving IL-1 blockade treatment. They found that renal functions were maintained or improved in 79.4% but deteriorated in 20.6% among 35 patients, not on dialysis (20). Data on pediatric FMF-related amyloidosis are insufficient in the literature. The study of Özçakar et al. examined six pediatric patients with FMF-related amyloidosis following 1 year of anakinra treatment, attacks completely disappeared or decreased in frequency, and partial remission occurred in patients with nephrotic syndrome. Anakinra treatment was initiated in three patients after renal transplantation, and their life quality improved (21).

Topaloglu et al. reported an improvement in inflammation findings and proteinuria after anti-IL-1 treatment in three patients with AID-related amyloidosis. However, the renal amyloid prognostic score did not improve in one patient. It even progressed in two patients, showing renal tissue damage after improving proteinuria with anti-IL-1 treatment in AID-related amyloidosis (22). Sözeri et al. reported that in a patient with FMF-related renal amyloidosis and nephrotic range proteinuria, 26-month canakinumab treatment resulted in improved inflammatory parameters and decreased proteinuria (23). Colchicine and biologic agents aiming at reduced SAA protein in FMF could facilitate the regression of amyloid accumulation (24). The study of Yildirim et al. included 18 patients with FMF-related amyloidosis (12 with preserved renal function, six with impaired renal function). All patients with preserved renal function had more than a 50% decrease in proteinuria at 12 months compared with baseline values, and none of the patients with impaired renal function had more than a 50% decrease in proteinuria. Canakinumab was not found to be effective in decreasing proteinuria in patients with FMF who already had impaired renal function (25).

The patient who was admitted with MAS findings had no infectious, malignant or immunologic etiology and was evaluated as having FMF-related MAS. As in our patient, case reports have shown that activation of the proinflammatory cytokine milieu may cause MAS in patients with undiagnosed FMF (26). FMF induces hyperactivation of the NLRC4 inflammasome and can result in MAS. NLRC4 triggers the inflammasome, an innate immune complex that responds *via* caspase-1 activation and IL-1β and IL-18 secretion (27, 28).

The most important limitations of our study were its retrospective design and relatively short duration of treatment time in terms of safety. The most important strength of our study was the high number of patients. This article evaluates the most significant number of pediatric patients with colchicine resistance or intolerant pediatric FMF using canakinumab in the literature.

In conclusion, our study showed that canakinumab treatment was highly effective, well-tolerated, and had a very low incidence of adverse effects in pediatric patients with FMF with colchicine resistance or intolerance. In addition, it was also shown that a controlled extension of the canakinumab dose interval in patients with FMF was safe. We did not observe any patient who developed amyloidosis with colchicine and canakinumab treatment in our study cohort. Potential efficacy of canakinumab treatment in preventing amyloidosis in FMF should be tested in long-term controlled trials.

## Data Availability Statement

The original contributions presented in the study are included in the article/supplementary materials, further inquiries can be directed to the corresponding author/s.

## Ethics Statement

The studies involving human participants were reviewed and approved by the ethics committee of Ondokuz Mayis University (No: OMUKAEK 2021/1). Written informed consent from the participants' legal guardian/next of kin was not required to participate in this study in accordance with the national legislation and the institutional requirements.

## Author Contributions

OA and BY designed the study. AY and BY collected and analyzed data. OA, BY, and HN wrote the manuscript. All authors read and approved the final manuscript.

## Conflict of Interest

The authors declare that the research was conducted in the absence of any commercial or financial relationships that could be construed as a potential conflict of interest.

## Publisher's Note

All claims expressed in this article are solely those of the authors and do not necessarily represent those of their affiliated organizations, or those of the publisher, the editors and the reviewers. Any product that may be evaluated in this article, or claim that may be made by its manufacturer, is not guaranteed or endorsed by the publisher.
